# Mutual Information as a General Measure of Structure in Interaction Networks

**DOI:** 10.3390/e22050528

**Published:** 2020-05-07

**Authors:** Gilberto Corso, Gabriel M. F. Ferreira, Thomas M. Lewinsohn

**Affiliations:** 1 Departamento de Biofísica e Farmacologia, Centro de Biociências, Universidade Federal do Rio Grande do Norte (UFRN), Natal-RN 59072-970, Brazil; 2 Departamento de Biologia Animal, C.P. 6109, Instituto de Biologia, Universidade Estadual de Campinas (UNICAMP), Campinas-SP 13083-970, Brazil; gabrielfelixmf@gmail.com (G.M.F.F.); thomasl@unicamp.br (T.M.L.); 3 Wissenschaftskolleg zu Berlin, 14193 Berlin, Germany

**Keywords:** interaction diversity, community ecology, specialization

## Abstract

Entropy-based indices are long-established measures of biological diversity, nowadays used to gauge partitioning of diversity at different spatial scales. Here, we tackle the measurement of diversity of interactions among two sets of organisms, such as plants and their pollinators. Actual interactions in ecological communities are depicted as bipartite networks or interaction matrices. Recent studies concentrate on distinctive structural patterns, such as nestedness or modularity, found in different modes of interaction. By contrast, we investigate mutual information as a general measure of structure in interactive networks. Mutual information (MI) measures the degree of reciprocal matching or specialization between interacting organisms. To ascertain its usefulness as a general measure, we explore (a) analytical solutions for different models; (b) the response of MI to network parameters, especially size and occupancy; (c) MI in nested, modular, and compound topologies. MI varies with fundamental matrix parameters: dimension and occupancy, for which it can be adjusted or normalized. Apparent differences among topologies are contingent on dimensions and occupancy, rather than on topological patterns themselves. As a general measure of interaction structure, MI is applicable to conceptually and empirically fruitful analyses, such as comparing similar ecological networks along geographical gradients or among interaction modalities in mutualistic or antagonistic networks.

## 1. Introduction

Entropy models and measures have been applied in a variety of areas in ecology, such as ecological genetics [1], macroecology [2], landscape ecology [3] and ecological economics [4]. Entropy-based models have been prominent especially in two fields: circulation models for ecosystems [5,6,7,8,9] and measurement of species diversity in communities. Ramón Margalef [10,11] pioneered the use of the Shannon–Weaver function to assess the diversity of collections with non-uniform species abundances [12]. This metric came into widespread use from the 1960s onwards, both because of its simplicity and the appealing possibility that it might represent the actual “information content” of multispecies assemblages [11,13], see [14,15].

More recently, two further developments rekindled interest in this metric: first, partitioning diversity within and among spatial units (alpha versus beta diversity, see, e.g., the Forum papers introduced by Ellison [16]) or among a hierarchy of spatial levels [17]. The second is the measurement of interactions between species in ecological networks, and is the subject for the current paper.

Ecological interactions span a wide range of modes of directional or reciprocal ways in which individuals, populations or species assemblages influence one another. Classical modes include mutualisms, i.e., mutually beneficial interactions, such as plant associations with their animal dispersers and pollinators [18] or with mycorrhizal fungi and soil bacteria [19]. Many mutualistic interactions are nontrophic, which do not involve consumption. By contrast, except for competition, most antagonistic interactions are trophic between resource and consumer taxa, such as prey and predators, or hosts and parasites or pathogens [20,21,22]; in combination, these make up food webs. Interactions may also switch from positive to negative effects, as in plant species that compete or facilitate coexistence under different circumstances [23].

The Shannon-Weaver entropy of a biotic assemblage—usually, a group of species belonging to a given taxon—measures the "information content" of that assemblage; in other terms, it measures the uncertainty of the species to which a randomly drawn individual belongs. Thus, entropy is higher in more diverse assemblages. By contrast, the mutual information of an interactive assemblage corresponds to the reduction in the uncertainty of the species affiliation of an individual randomly drawn from the assemblage, if we know the identity of the individual(s) with which it interacts. Therefore, mutual information is higher in assemblages with a higher degree of specialization, in which individuals (and species) have fewer interaction partners. In bipartite networks, mutual information will be maximal when reciprocal specialization is maximal; in the special case of square matrices, all relationships will be biunique or bijective (for instance, each resource species is consumed by only one consumer species and vice versa).

Recent investigations of interaction patterns in ecological communities have been strongly stimulated by developments of complex network theory [24,25]. Subsequently, ecological research has favoured network depictions and analyses. However, bipartite networks can be depicted and analyzed equally well as matrices or as multivariate systems in ordination space [26]. To examine entropy and its components in interactive systems we represent them here as bidimensional matrices, with cross-references to their equivalents in network terminology that are widely used in the current ecological literature.

In this paper, we scrutinize the effect of an array of matrix (or network) attributes on mutual information, starting from primary or first-order parameters of simple patterns [27] and progressing to more complex structures, notably topologies which have been intensively investigated by ecologists in recent years [26,28]. Our goal is to evaluate whether mutual information is suitable for general comparisons of structure among interaction matrices, whatever their interaction modality, dimensions and topological attributes.

A word of caution about the computation of entropy or complexity in networks. In fact, several network entropy measures have been developed in the literature, see for instance [29]. In addition, entropy measures are dependent on probability distribution associated to the graph, for instance, connectivity, distances, or clustering distributions, but a unique entropy measure for the network is a fragile concept [30]. In this study, we do not deal with a global entropy or information measure for a network, but rather to the mutual information between the two marginal sets that constitute a bipartite network.

## 2. Mutual Information—Setting the Problem

The mathematical notation we employ is based on a widely-used source [31]. We consider two sets *X* and *Y* that represent two sets of biological entities, linked by a given mode of biotic interaction, such as consumer and resource species in a community. The entropy of these sets is H(X) and H(Y). The formal definition of entropy comes from the original Shannon paper [32]:

(1) $$H\left(X\right)=\;-\sum_{i=1}^{N_{X}}p_{i}\;log\;p_{i}$$

where $p_{i}$ are the probabilities associated with the $N_{X}$ states in which the elements of the set *X* are distributed. In community ecology $1\leq i\leq N_{X}$ represents species *i* and $N_{X}$ the total number of species in the *X* subset of the community and, correspondingly, *j* and $N_{Y}$ for the species in the *Y* community subset. The elements of sets *X* and *Y* are functionally connected, forming a web of interactions. This web, in fact a bipartite network, is defined by an adjacency matrix $A=a_{i,j}$.

In the special case of the entropy in which the distribution is flat, $p_{i}=1/N_{X}$ for all *i* species of the $N_{X}$ set. The entropy in this case is:

(2) $$H\;=\;-\sum_{i=1}^{N_{X}}\frac{1}{N_{X}}\;log\;\frac{1}{N_{X}}\;=\;logN_{X}$$

It is possible to prove that the limit of this equiprobable distribution corresponds to the maximal entropy [31,32].

Next, we formalize the concept and notation for interacting populations and networks. For the set *X* the population species distribution is the number of individuals belonging to species *i*, i.e., $k_{i}\left(X\right)$ with $1\leq i\leq N_{X}$, in the $N_{X}$ species that form the *X* subset. Likewise, $k_{j}\left(Y\right)$, with $1\leq j\leq N_{Y}$, is the distribution of individuals in the $N_{Y}$ species that form the *Y* interactive subset. Both $k_{i}\left(X\right)$ and $k_{j}\left(Y\right)$ can be computed from the matrix adjacency elements $a_{i,j}$:

(3) $$k_{i}\left(X\right)\;=\;\sum_{j=1}^{N_{Y}}a_{i,j}\;k_{j}\left(Y\right)\;=\;\sum_{i=1}^{N_{X}}a_{i,j}$$

From $k_{i}\left(X\right)$ we compute $p_{i}\left(X\right)=\frac{k_{i}\left(X\right)}{N_{M}}$ for $N_{M}=\sum_{i,j}a_{i,j}$, the total number of interactions in the community; similarly, $p_{j}\left(Y\right)=\frac{k_{j}\left(Y\right)}{N_{M}}$. For $p_{l}\left(X,Y\right)=\frac{a_{l}}{N_{M}}$ the probability matrix, we assume that $a_{l}$ is the conversion of the matrix $a_{i,j}$ into a vector of size $N_{L}$ (the number of interactions of the network), and $p_{l}\left(X,Y\right)$ is a probability associated with the interaction matrix. Finally, H(X), H(Y) and H(X,Y) are computed from $p_{i}\left(X\right)$, $p_{j}\left(Y\right)$ and $p_{l}\left(X,Y\right)$, respectively.

According to references [31,33] the mutual information I(X;Y) of two interconnected sets *X* and *Y* can be obtained from the entropies of the two sets and the joint entropy of both sets H(X,Y):

(4) I(X;Y)=H(X)+H(Y)−H(X,Y)

In set theory, H(X,Y) is the entropy of the joint set, or union set, and I(X;Y) the entropy of the intersection set between *X* and *Y*. A normalized version *S* of mutual information [33] is given by:

(5) $$S\;=\;\frac{I(X;Y)}{H(X,Y)}$$

The quantity *S* has a range of 0≤S≤1 which allows comparing networks of different sizes, one of the goals of the present paper.

**Figure 1:**
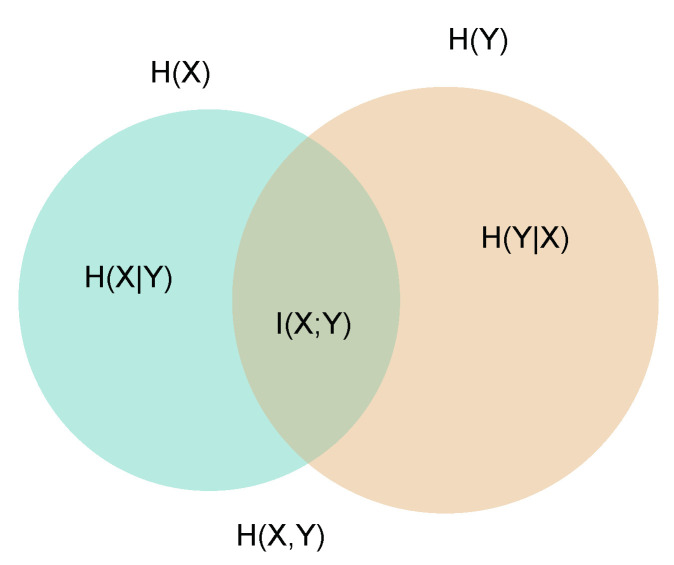
Venn diagram representing two interconnected subsets *X* and *Y* with their entropies, *H(X)* and *H(Y)*, their joint entropy *H(X,Y)* and their mutual information, *I(X;Y). H(X|Y)* is the conditional entropy of *X* given *Y*, and *H(Y|X)* its converse.

Note that H(X,Y) is maximal when there is no overlap between the sets H(X) and H(Y) in Figure 1,

(6) $$H{\left(X,Y\right)}_{max}\;=\;H\left(X\right)+H\left(Y\right)$$

representing the scenario where X and Y are independent sets, and the values in each matrix cell $a_{i,j}$ is directly proportional to the marginal probabilities $p_{i}\left(X\right)$ and $p_{j}\left(Y\right)$. In ecological terms, H(X,Y) is maximal in the absence of specialization, when species interact proportionally to their abundances.

By combining Equations (4) and (6), it is possible to show that $H_{2}^{\prime }$, a widely used metric of specialization in ecological networks [34], is the mutual information normalized by its maximal value, given the marginal totals of the matrix:

(7) $$H_{2}^{\prime }\;=\;\frac{H{\left(X,Y\right)}_{max}-H\left(X,Y\right)}{H{\left(X,Y\right)}_{max}-H{\left(X,Y\right)}_{min}}$$

(8) $$H_{2}^{\prime }\;=\;\frac{I(X;Y)}{I{\left(X;Y\right)}_{max}}$$

In the next section we explore a sequence of simple models that can be solved analytically and/or compared with exact ones. Analytical results were tested on artificial models in the R-program environment [35].

## 3. Baseline Models

### 3.1. Uniform Networks

**Figure 2:**
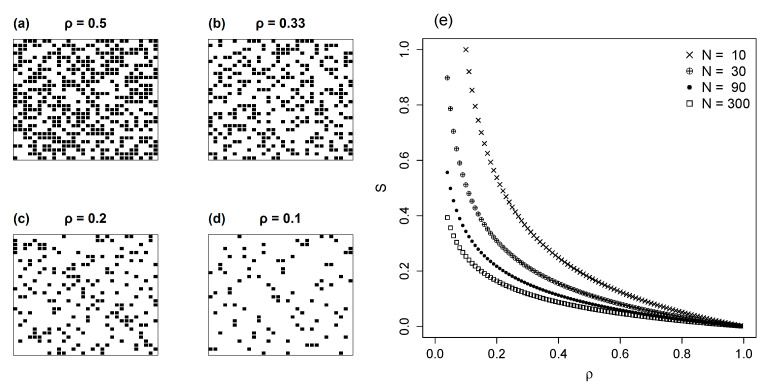
Square matrix with uniform distributions; (**a**–**d**) show representative patterns for levels of occupancy, ρ, from 0.1 to 0.5. (**e**) shows values of *S*, solved by Equation (9), plotted against the full range of ρ, for matrices varying in size ($N=N_{X}=N_{Y}$).

Our initial model is a square lattice in which $N_{X}=N_{Y}=N$, with randomly filled sites with a constant $k_{i}$. The occupancy ρ is defined as the ratio between the total number of actual interactions in the matrix $N_{M}$ and the total number of cells $N^{2}$, or $\rho =\frac{N_{M}}{N^{2}}$. Figure 2a–d illustrates four cases of matrices with uniform distribution and decreasing ρ.

By assuming that occupancy ρ follows a flat distribution, the entropy is maximal. Following Equation (2) we have H(X)=H(Y)=logN. To compute the entropy of the matrix we employ Equation (2) with number of states $\rho \;N^{2}$. Using Equation (5) we compute the normalized mutual information:

(9) $$S\;=\;\frac{2\;log\;N-log\;\rho N^{2}}{log\;\rho N^{2}}\;=\;\frac{log\;\frac{1}{\rho }}{log\;\rho N^{2}}$$

In Figure 2e we plot *S* versus ρ, for different numbers of species *N*. As expected from Equation (9), for a given ρ, mutual information decreases with *N*. In fact $S\propto {\left(logN^{2}\right)}^{-1}$. Figure 2e also shows that S→0 as ρ→1.

### 3.2. Random Networks

**Figure 3:**
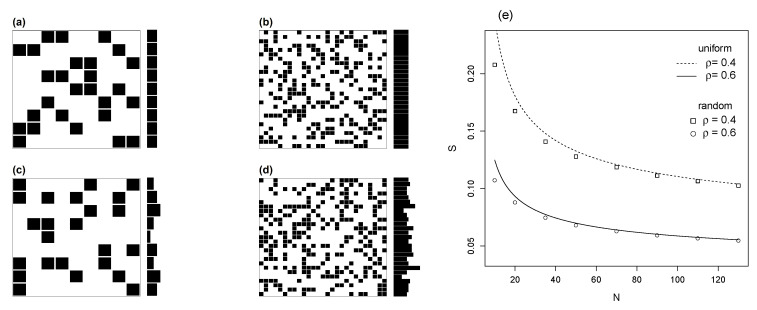
Uniform versus random matrices; comparison between the exact solution of the uniform model, Equation (9), and a Monte Carlo random model. In the uniform model (**a**,**b**) the marginal totals (represented by the bars to the right of the matrices) are held constant, whereas in random models (**c**,**d**), marginal totals vary freely; only occupancy for the entire matrix, ρ, is held constant. Figure (**e**) shows mutual information versus matrix size for both cases, with ρ=0.4 and 0.6. The uniform model Equation (9), is represented by lines and the Monte Carlo random model by symbols; see insert legend.

Random networks are the most important class of networks for null models in theoretical ecology [36]. The construction of proper random networks as null models for bipartite networks is a theme of longstanding debate [37]. Any random model is associated to a distribution of probabilities. The random network with fixed $k_{i}$ is the uniform or planar network presented before; this is a convenient starting point because it assumes no a priori information on the classes within the interconnected sets *X* and *Y*. In random models $k_{i}$ is not strictly fixed, so that marginal totals can vary within the bound of total occupied cells, $\rho {\left(N\right)}^{2}$. Thus, the random matrix is a relaxed version of the uniform model; compare Figure 3a with Figure 3c and Figure 3b with Figure 3d.

The random models we employ are Monte Carlo randomizations with total occupancy ρ fixed, but marginal totals free to vary. Figure 3e shows that for large size *N* the results coincide. This is expected, since for a large number of species the uniform model and the random model are quite close. On the other side, for smaller *N* (here, with *N* approximately ≤ 50), the *S* of the uniform model slightly exceeds the random model.

### 3.3. Matrix Shape

The preceding square network models fixed the same number of classes (species) in both interactive subsets. We call networks with X≠Y size-asymmetrical; for non-square matrices, matrix shape is expressed as $N_{X}/N_{Y}$. In order to test the effect of matrix shape on mutual information, we expand the square uniform model of Section 3.1 for distinct $N_{X}$ and $N_{Y}$ instead of a common *N*. A general formula for *S* is developed as in previous Equation (9). Here, mutual information is written as:

(10) $$S\;=\;\frac{log\;N_{X}+log\;N_{Y}-log\;\rho N_{X}N_{Y}}{log\;\rho N_{X}N_{Y}}\;=\;\frac{log\;\frac{1}{\rho }}{log\;\rho N_{X}N_{Y}}$$

From Equation (10) we see that if the product of $N_{X}$ by $N_{Y}$ is constant, then *S* is the same as when $N_{X}=N_{Y}=N$. For instance, a matrix with sizes $N_{X}=40\;,\;N_{Y}=40$ will have the same *S* as a matrix of sizes $N_{X}=80\;,\;N_{Y}=20$. This is in fact the most important result for matrix shape. Note, however, that whereas S is invariant for matrices of any shape given their total size, occupancy is constrained by shape. For a non-degenerate square matrix the minimum ρ implies in $N=N_{X}=N_{Y}$ which is attained in diagonal matrices. At the other extreme, to be non-degenerate a same-sized linear matrix ($N_{X}=1$ or $N_{Y}=1$) requires maximum occupancy, ρ=1.

## 4. Simple Topologies

### 4.1. Nested Networks

In this section we compute mutual information for nested networks. First, we take as baseline model the square matrix with all cells above the diagonal line occupied, for which we provide an exact solution. Second, the general case with any occupancy is explored by Monte Carlo simulation.

**Figure 4:**
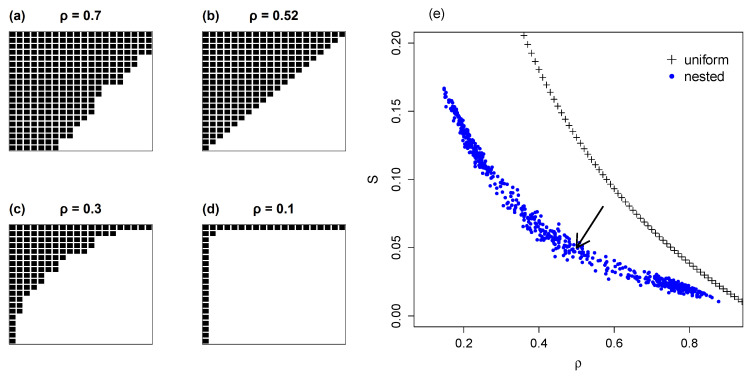
Mutual information versus occupancy for nested matrices, with N=20. Figures (**a**–**d**) show nested matrices with decreasing occupancies.; Plot (**e**) shows the normalized mutual information *S* of a set of nested matrices of distinct occupancies. We plot also *S* of the uniform model for comparison. The analytical result of Equation (14) for half occupancy (**b**), is indicated with an arrow.

In the initial model the square matrix is symmetric: $N_{X}=N_{Y}=N$, so that H(X)=H(Y). Also, ρ is always above 0.5; as N→∞, ρ→0.5. This model is illustrated in Figure 4b; according to usage in the ecological literature, nesting is represented from cell $a_{1,1}$ outwards, so that the matrix is packed above the antidiagonal.

The number of interactions of each species is given by $k_{i}=i$. The interaction probability of each species is normalized as $p_{i}=i/N_{M}$, scaling it by $N_{M}$, the sum of all realized interactions for this half-occupied matrix:

(11) $$N_{M}=\sum_{i=1}^{N}i=\frac{N(N+1)}{2}.$$

From the species interaction probabilities we compute the species entropy using the usual definition, Equation (1):

(12) $$H\left(X\right)\;=\;-\sum_{i=1}^{N}\frac{i}{N_{M}}log\;\frac{i}{N_{M}}\;=\;\sum_{i=1}^{N}\frac{i}{N_{M}}\left(log\;N_{M}-log\;i\right)$$

Using the hyperfactorial function Hyp(x) (see Appendix A) we obtain:

(13) $$H\left(X\right)\;=\;log\;N_{M}-\frac{1}{N_{M}}log\;Hyp\left(N\right)$$

To compute *S* we need to estimate first the total matrix entropy H(X,Y), given the total number of interactions $N_{M}$, $H\left(X,Y\right)=log\left(N_{M}\right)$. From Equation (5) we then estimate the normalized mutual information:

(14) $$S\;=\;\frac{2\left(log\;N_{M}-\frac{1}{N_{M}}log\;Hyp\left(N\right)\right)-log\;N_{M}}{log\;N_{M}}$$

The general case of nested networks is explored with an algorithm that computes nested matrices with any size and occupancy. The mutual information of randomly generated nested matrices was computed with Equation (5).

Through Monte Carlo simulation we produced 200 samples with variable occupancies in square matrices of size N=20. Figure 4e shows the distribution of normalized mutual entropy versus ρ. The analytical result of Equation (14) for the special case of a half-occupied matrix is indicated by the arrow; in this case ρ≈0.5 corresponds to S≈0.05. The value of S increases for small ρ, up to the limit of one line and one column occupied (see Figure 4d). Conversely, as ρ→1, S→0 as seen before (Section 3.1). The chief result of the comparison in Figure 4e is that nested matrices always have a smaller mutual information than matrices with uniform marginal distributions of same dimension and occupancy, due to their difference in degree distributions.

### 4.2. Isometric Modules

**Figure 5:**
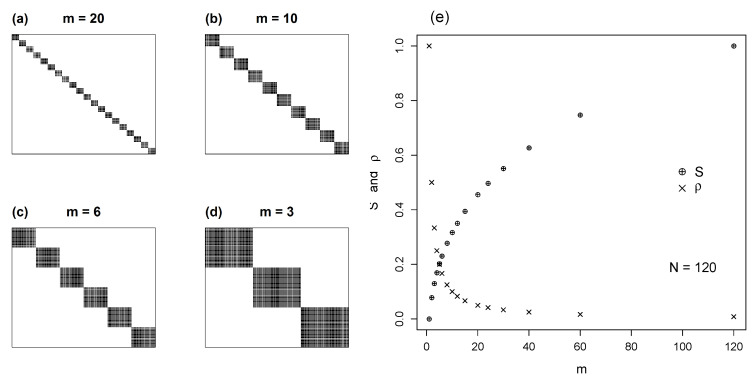
Models with isometric modules in square matrices of size N=120. Panels (**a**–**d**) show four cases varying from m=3 to 20. Panel (**e**) shows the relation of mutual information *S* and occupancy ρ with the number of modules *m*. The limit case m=120 corresponds to the diagonal matrix, whereas m=1 corresponds to ρ=1 and S=0. The graph also represents the expected inverse relation between ρ and *S*.

The second topology of interest to ecologists is modularity or compartmentation, which is often found in interaction networks. As before, we examine a square matrix of size *N* in which interactions are set out as *m* equal modules of size *t*. Figure 5 illustrates this model in square matrices of size *N*, varying from m=20 and t=6, to m=3 and t=40. Note that when t=1, m=N, the diagonal matrix which is the limit case of H(X)=H(Y)=H(X,Y)=I(X;Y).

Because all modules are of equal size, the species distributions in *X* and *Y* are trivially flat. Therefore, we can use the previous result (Equation (2)), to compute *S*. The number of species is given by $N_{X}=N_{Y}=m\;t$, and the number of realized interactions in the matrix is $m\;t^{2}=N\;t$. Combining these, we obtain:

(15) $$S\;=\;\frac{log\;N+log\;N-log\;N\;t}{log\;N\;t}\;=\;\frac{log\;N^{2}/N\;t}{log\;N\;t}$$

or

(16) $$S\;=\;\frac{log\;N/t}{log\;N\;t}\;=\;\frac{log\;N-log\;t}{log\;N+log\;t}$$

Figure 5e shows how mutual information and occupancy vary with the number of modules in isometric modular matrices. *S* and ρ are, respectively, increasing and decreasing functions of the number of modules. The inverse relation between *m* and ρ is also quite simple. As ρ is the ratio between the number of occupied cells and the total cell number, it follows that:

(17) $$\rho \;=\;\frac{mtt}{NN}=\;\frac{mtt}{mt\;mt}=\;\frac{1}{m}$$

For example, m=2 corresponds to ρ=0.5 and m=3 to ρ=0.333 (Figure 5e).

It is also relevant to explore when two modular matrices with different number of modules and different sizes will have identical normalized mutual information. In other terms, what are the conditions for matrices $M_{1}$ and $M_{2}$ with, respectively, $m_{1}$ and $m_{2}$ numbers of modules and $t_{1}$ and $t_{2}$ module sizes to have identical *S*. Since we are only considering square matrices with isometric modules, the number of species in the matrices are, respectively, $N_{1}=N_{X_{1}}=N_{Y_{1}}$ and $N_{2}=N_{X_{2}}=N_{Y_{2}}$. From Equation (16), the condition of equal *S* for two modular matrices is:

(18) $$\frac{log\;N_{1}-log\;t_{1}}{log\;N_{1}+log\;t_{1}}=\;\frac{log\;N_{2}-log\;t_{2}}{log\;N_{2}+log\;t_{2}}$$

Algebraically, Equation (18) is equivalent to:

(19) $$\frac{log\;N_{1}}{log\;t_{1}}\;=\;\frac{log\;N_{2}}{log\;t_{2}}$$

Using basic logarithm properties, the equation above can be rewritten in a more intuitive way as:

(20) $$log_{\;t_{1}}N_{1}\;=\;log_{\;t_{2}}N_{2}$$

**Figure 6:**
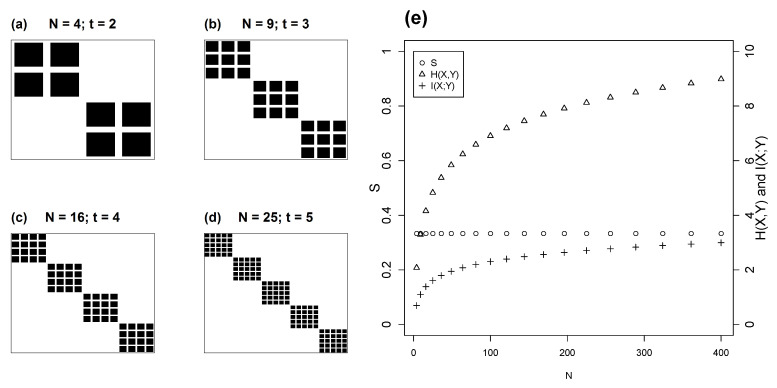
Square matrices with equal-size modules. Matrix size, number of modules and module size increase from (**a**–**d**). Plot (**e**) shows normalized mutual information *S*, mutual information I(X;Y), and total entropy H(X,Y) plotted against increasing matrix size, while keeping $log_{t}N$ constant

Thus, according to Equation (20), two isometric modular matrices will have the same S if, and only if, the number of species by module and the number of total species in the matrix are in the same power relationship in both matrices. To illustrate Equation (20) we show in Figure 6a–d cases that share the same *S*. In Figure 6e we explore the behaviour of *S*, H(X,Y) and I(X;Y) when matrix size (*N*) increases while keeping $log_{t}N$ constant. Figure 6e shows that *S* remains constant in this case because both H(X,Y) and I(X;Y) increase by the same proportion.

### 4.3. Non-Square Modular Matrices

The last simple model we examine is the case of non-square modular matrices. Given that modules are identical, they are also non-square and the size of each of the *m* modules is represented as *t* by *z*. Therefore, the number of species in the *X* set is $N_{X}=m\;t$ and in the *Y* set $N_{Y}=m\;z$.

As before, the two distributions of *X* and *Y* sets are flat, so we can again apply Equation (2). Following Equation (15) we obtain:

(21) $$S\;=\;\frac{log\;m\;z+log\;m\;t-log\;m\;t\;z}{log\;m\;t\;z}\;=\;\frac{log\;m}{log\;m\;t\;z}$$

Note that Equation (21) is a special case of Equation (19). Indeed, Equation (19) becomes (21) for t=z and N=mt.

Furthermore, the condition for two asymmetrical matrices with $m_{1}$, $t_{1}$, $z_{1}$ and $m_{2}$, $t_{2}$, $z_{2}$ parameters to have the same normalized mutual information is given by:

(22) $$\frac{log\;m_{1}}{log\;m_{1}\;t_{1}\;z_{1}}\;=\;\frac{log\;m_{2}}{log\;m_{2}\;t_{2}\;z_{2}}$$

## 5. Complex Topologies

In this section we advance beyond simple models of bipartite networks. Among many ways of producing more complex structures, we focus on two that are especially relevant to interactions in actual ecological assemblages.

### 5.1. Modules of Varying Size

**Figure 7:**
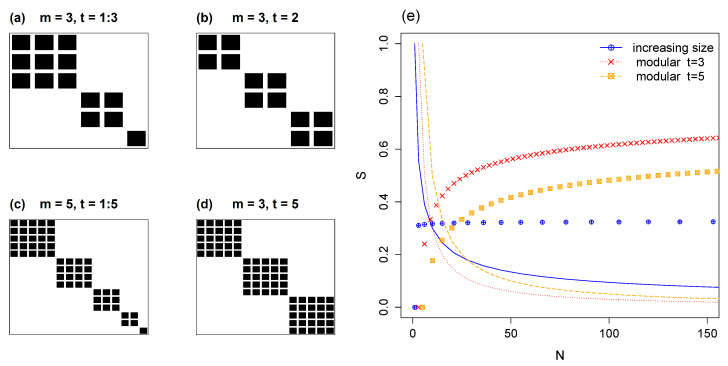
Matrices with varying module size. Panels (**a**,**c**) show modules of increasing size for m=3 and m=5, respectively; for comparison, panels (**b**,**d**) illustrate m=3 modules of constant size. Panel (**e**) shows *S* versus size *N* for modules of increasing size, compared with the case of constant size for two distinct *t*. The corresponding occupancy is indicated with lines.

In this subsection we consider a model composed of square modules of increasing size. Two examples are given in Figure 7; in (a), a matrix of size N=6 with three modules of size *t* = 1, 2 and 3; in (c), N=15 and five modules with sizes *t* = 1 to 5. For the general case with *m* modules the number of species $N_{X}=N_{Y}=N$ is given by:

(23) $$N\;=\;\sum_{i=1}^{m}i=\frac{m(m+1)}{2}$$

The total number of interactions in the network is:

(24) $$N_{M}\;=\;\sum_{i=1}^{m}i^{2}=\frac{m(m+1)(2m+1)}{6}$$

From the combination of Equations (23) and (24) we calculate the occupancy ρ of this model as a function of the number of modules *m*:

(25) $$\rho =\frac{N_{M}}{N^{2}}=\frac{\frac{m(m+1)(2m+1)}{6}}{{\left(\frac{m(m+1)}{2}\right)}^{2}}=\frac{2\;(2m+1)}{3m\;(m+1)}$$

Given the species probabilities $p_{i}=\frac{i}{N_{M}}$, for *i* as the number of species in each module, we compute entropy using Equation (1):

(26) $$H\left(X\right)\;=\;-\sum_{i=1}^{m}i\frac{i}{N_{M}}log\;\frac{i}{N_{M}}\;=\;\sum_{i=1}^{m}\frac{i^{2}}{N_{M}}\left(log\;N_{M}-log\;i\right)$$

Using the hyperfactorial squared function $\hat{Hyp}\left(x\right)$, detailed in Appendix A, we obtain:

(27) $$H\left(X\right)\;=\;\frac{N_{M}\;log\;N_{M}-log\;\hat{Hyp}\left(m\right)}{N_{M}}$$

From Equation (27) we then calculate the normalized mutual information, Equation (5), as:

(28) $$S\;=\;\frac{2H\left(X\right)-log\;N_{M}}{log\;N_{M}}$$

To assess mutual information in models with increasing modules, they are compared with their counterparts (same *N*) with constant-sized modules, in which *S* depends only on *m* and *t*. Thus, results of Section 4.2 are compared with Section 5.1. In Figure 7e, normalized entropy *S* is plotted against *N*, with each point corresponding to a distinct *m*. The occupancies ρ corresponding to each *S* curve are plotted as continuous lines with a common range of zero to one. Notably, the normalized mutual information remains almost constant if we expand the matrix by adding modules of increasing size (Figure 7e). Despite the positive effect on *S* of decreasing the matrix’s occupancy (see Section 3.1), this is offset by the negative effect on *S* of increasing the degree unevenness of the matrix (Section 4.1).

### 5.2. Compound Models with Nested Modules

**Figure 8:**
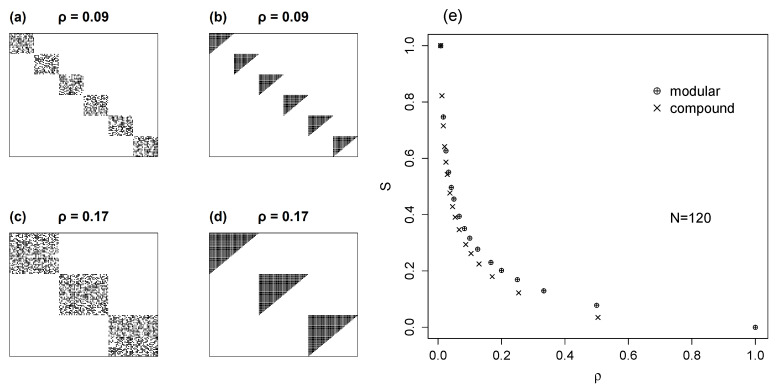
Examples of two simple modular matrices (**a**,**c**) and two compound matrices (**b**,**d**) with similar parameters. Plot (**e**) shows mutual information for various levels of occupancy in these two topologies. Mutual information of simple modules calculated with Equation (16), of compound matrices with Equation (32). Note that, for any given ρ, matrices with compound topology have smaller *S* than their simple modular counterparts.

The final model that we consider is the combination of two topologies. This model is hierarchical: a modular structure whose modules are internally nested. This compound topology is of special interest because it accords with common features of ecological and evolutionary scenarios [26,38,39]. To examine mutual information in these compound models, as before we set up square matrices with *m* internally nested modules. This case is show in Figure 8b for the particular case of m=4. Because of matrix symmetry, $N_{X}=N_{Y}=N$ and H(X)=H(Y). Each isometric module has $\frac{N}{m}=t$ species, and the total number of interactions in each module is given by $K_{m}$:

(29) $$K_{m}=\sum_{i=1}^{t}i=\frac{t\left(t+1\right)}{2}$$

The total number of interactions in the networks is $N_{M}=m\;K_{m}$. The entropy of the matrix rows (or columns) is computed summing the entropies over the *m* modules:

(30) $$H\left(X\right)\;=\;-m\sum_{i=1}^{t}\frac{i}{N_{M}}log\;\frac{i}{N_{M}}\;=\;\sum_{i=1}^{t}\frac{i}{N_{M}}\left(log\;N_{M}-log\;i\right)$$

Using the hyperfactorial function $\hat{Hyp}\left(x\right)$, introduced in the previous section, we have a closed form for the Equation (30) entropy:

(31) $$H\left(X\right)\;=m\left(K_{m}\;log\;N_{M}-\frac{1}{N_{M}}log\;Hyp\left(t\right)\right)$$

The normalized mutual information, Equation (5), is then:

(32) $$S\;=\;\frac{2H\left(X\right)-log\;N_{M}}{log\;N_{M}}$$

Equation (32) presents the analytical solution for mutual information in matrices with internally nested modules. To compare these with simple modular matrices (Section 4.2), we set a common size (N=120) for the square matrices, and a similar occupancy, for both topologies; compare Figure 8a with b, and c with d. In Figure 8e, mutual information is plotted against occupancy which, as seen before, is an inverse function of number of modules (Equation (17)).

As Figure 8e shows, compound matrices have smaller values of normalized mutual information *S* than the corresponding simple modular matrices. This result is in agreement with the difference between uniform, Section 3.1 and nested networks seen in Section 4.1. In fact, both plots, Figure 8e and Figure 4e, indicate the same result: nested patterns have less reciprocal information than matrices with uniform link distributions.

## 6. Does Mutual Information Vary with Topology?

The results of the previous sections suggest that mutual information is sensitive to network topology. However, is this sensitivity to topology due solely to the effects of lower-order network properties [27] on MI? How does MI change if we alter the matrix topology while holding size, occupancy and degree distribution constant ?

**Figure 9:**
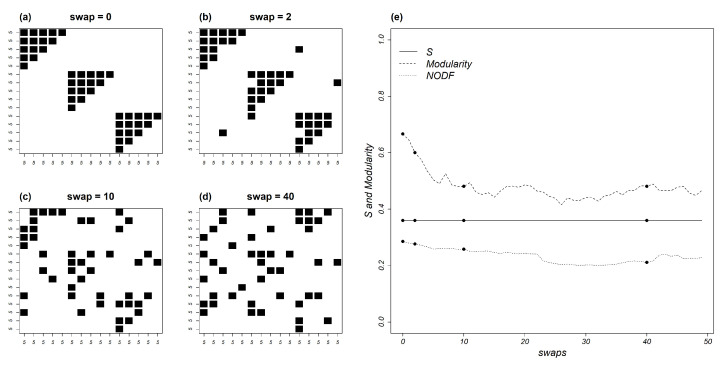
A compound matrix with identical internally nested modules (**a**) whose topology is disrupted by sequential swaps, (**b**–**d**). Panel (**e**) shows the effect of the swap sequence on normalized mutual information (*S*), modularity, measured according to [41], and nestedness, measured by NODF [42]. Whereas both components of the compound pattern are disrupted by swapping, mutual information remains constant.

To address this question we set up a square matrix, N=15, composed of equally sized square nested modules, t=5 and m=3 as in Figure 9a. We then apply sequential swaps [40] on this matrix (Figure 9b–d) altering its structure while holding its occupancy and its degree-distribution constant. As shown in Figure 9e, the swaps decrease both network modularity (which is disrupted within circa 10 swaps, Figure 9c), and nestedness. However, mutual information is completely unaffected.

This is a robust demonstration that, for a given set of lower-order network parameters [27], mutual information is completely insensitive to changes in topology. Since mutual information is closely connected to specialization, this conclusion has profound implications for the relationship between specialization and topology. The degree of specialization of a network sets the space of topologies possible for that network, and, conversely, any topology within this space is bound to that level of specialization. No variation in topology is possible at both extremes, either MI=1 (maximum specialization) and MI=0 (minimum specialization). Variation in topology is possible along the continuum 0<MI<1, with maximal freedom at MI=0.5.

## 7. Discussion and Conclusions

The chief goal of this study was to assess the suitability of mutual information as the basis for a general measure of reciprocal correspondence in a bipartite set of interacting entities, notably biological species. On theoretical grounds, MI is arguably the simplest and most general way of representing such correspondence (Figure 1). However, several variables that set the structure of interactive matrices can potentially alter MI. Since we seek a metric applicable to interactive matrices of any kind, irrespective of their topologies and dimensions, sensitivity to structural differences would compromise the wide-ranging comparisons for which the metric is intended. In this respect, the present study differs from most explorations of metrics of interactive matrices, whose goal is to find descriptors indicative of particular topologies and patterns [34,42,43].

We set up a sequence of matrix models starting from the simplest ones in which we examined effects of various parameters on MI, providing analytical formulations whenever feasible for square and non-square matrices. We then inspected how MI behaves in the most commonly investigated topologies: nested or modular networks, and their combinations. Normalized mutual information is inversely correlated with matrix occupancy and with matrix size, as set by its formula (Figure 2). This relationship holds for matrices with uniform as well as random marginal distributions, although the actual values of S diverge in smaller matrices (Figure 3).

In nested interactive matrices, MI changes with occupancy as do simple non-structured matrices. This is shown in Figure 4, which also demonstrates that fully packed nested matrices have lower values of S than same-sized matrices with uniform degree distributions. This difference diminishes of course in saturated matrices. As in all interactive systems when ρ→1, S→0. In a community composed only of generalists, uncertainty on partnerships or associate species is maximal and reciprocal information is null.

Modular networks have a two-tiered structure, so that their topologies require at least one additional parameter. Using again the simplest possible model as starting point, we examined how module size and number affect MI. With isometric modules in a square matrix of a given size, MI is inversely correlated with the number of modules (Figure 5), which, in turn are inversely correlated with module size. In Figure 5a–d, we illustrate that occupancy increases as fewer and larger modules fill the interaction matrix. As seen in Figure 5e, MI is inversely related to occupancy. Hence, occupancy, given the network dimensions, arises as the key determinant of MI, irrespective of network topologies.

The choice of MI as a fundamental descriptor reflects our focus on interaction diversity. Most of the effort in modeling and parametrizing diversity and its components to date has been concerned with measuring species assemblages of a given taxon, such as birds or flowering plants. There are formal parallels between assessing entropy within bipartite networks and within the distribution of species over geographical space, especially when these are scored in discrete units such as islands or disjunct sampling sites. However, similar computation should not obscure essential differences. Processes which give rise to structure are very distinct in interactive networks as compared to biogeographical assemblages. Biotic assemblages adjust to, but do not essentially modify, the geographic units in which they exist. By contrast, interactions between two sets of species are organized according to the interaction modality, and derive from evolutionary and ecological processes which modify both species sets, each of which can have major effects on the other [44].

Entropy measures are also applied in vegetation ecology. As in interaction networks, in this case the distribution of plant species in vegetation units is a problem of reciprocal information, expressed as mutual information [43]. Nevertheless, the classification of plant communities is largely based on species composition; hence mutual information can only be used to compare classifications erected by researchers, presuming that more effective classifications will have higher mutual information.

The measurement of structure in interactive networks is closest to studies which address specialization, either one-way or reciprocal [34,45]. Specialization is a key process as well as a central component of interactive communities, both trophic and non-trophic [18,46,47]. By no coincidence, a widely-used measure of specialization, Blüthgen’s $H_{2}^{\prime }$ [34] is based on the Kullback–Leibler distance and its derivation is similar to our rationale. However, $H_{2}^{\prime }$ is distinguished by its denominator: realized interactions are normalized by the potential range of $H_{2}$ values given the matrix dimensions, whereas we normalize mutual information as a fraction of total matrix entropy. This reflects the different goals of these metrics. Blüthgen’s $H_{2}^{\prime }$ is intended to assess and compare specialization in communities that vary in their network topology and in other ecological attributes. In turn, we propose to compare the structure of interactive networks of any kind, regardless of their specific topologies. Scaling reciprocal information by total matrix entropy seems both conceptually and practically more appropriate to the latter goal.

The simple models that were examined in this paper, both analytically and through Monte Carlo procedures, help to elucidate the relationships of network dimensions and topologies and with mutual information as mediated by specialization. Neither modularity, nestedness or any other topological descriptor are direct measures of specialization. Instead, they describe how interactions are structured within a network that has a given degree of specialization (Figure 9). In this respect, a noteworthy result is the robustness of mutual information to compound as well as simple topologies. This is convincingly demonstrated by its invariance at any level of topology-destroying swaps (Figure 9).

In actual networks, sampling effects require consideration. Further sampling may add unrecorded species and/or interactions. Novel species are likely to be associated with a single partner, therefore increasing mutual information. Conversely, a novel interaction between previously recorded species will decrease mutual information. Thus, the effect of additional sampling on perceived network structure depends on whether interactions accumulate at a higher rate than collector’s curves for the interacting species in the assemblage.

Local bipartite interactive networks may be extended profitably in several ways, some of which we highlight here for further work. First, the orthogonal extension of a local interaction network over space (or time); three-dimensional interaction matrices include three two-way and one three-way mutual information components [43]. This richer model allows assessing whether, and how, MI shifts across space or, in other terms, whether it is invariant among local communities despite eventual spatial turnover of species. This can be assessed by comparing accumulation curves for species and for interactions among localities, as noted in the preceding paragraph.

Second, mutual information deserves investigation in multitrophic systems, starting with tritrophic models that overlay two interactive interfaces (such as plants, herbivores and predators or parasites). These are especially promising to explore whether mutual information matches or shifts noticeably between these interfaces. Alternatively, one set of species may interact in different modes with two other sets; for instance, plants with pollinators and with herbivores. Here, mutual information will be compared between two networks with one partner set in common. Different interaction modes, particularly mutualistic and antagonistic ones, have been contrasted in comparative analyses, searching especially for differences in specialization and topologies (e.g., [28]). However, few if any studies have investigated interactive networks with a shared species set. These are prime items in an agenda for promising developments of this growing field of ecological research.

## Appendix A. The Hyperfactorial Function

The factorial n! of a natural number *n*, is the product of all the integers up to *n*:

(A1) $$n!\;=\;1.2.3....n\;=\;\prod_{i=1}^{n}i$$

The factorial function is closely related to the sum of logarithm of integers. In fact, since the logarithm of a product of two numbers is equal to the sum of their logarithms,

(A2) $$\sum_{i=1}^{n}log\;i\;=\;log\left(\prod_{i=1}^{n}i\right)\;=\;log\;n!$$

Moreover, to compute the entropy of a nested matrix (Section 4.1), we obtain:

(A3) $$\sum_{i=1}^{n}\;i\;log\;i\;=\;\sum_{i=1}^{n}\;log\;i^{i}\;=\;log\left(\prod_{i=1}^{n}i^{i}\right)$$

In the above equation we arrive at the hyperfactorial function Hyp(n) defined as:

(A4) $$Hyp\left(n\right)\;=\;\prod_{i=1}^{n}i^{i}=1^{1}2^{2}3^{3}\;...\;n^{n}$$

In addition, the computation of the entropy of a modular matrix with increasing modules (Section 5.1) results in:

(A5) $$\sum_{i=1}^{n}\;i^{2}\;log\;i\;=\;\sum_{i=1}^{n}\;log\;i^{i^{2}}\;=\;log\left(\prod_{i=1}^{n}i^{i^{2}}\right)$$

We designate this new function the hyperfactorial squared function, $\hat{Hyp}\left(n\right)$, which is given by:

(A6) $$\hat{Hyp}\left(n\right)\;=\;\prod_{i=1}^{n}i^{i^{2}}$$
